# A hybrid scheduling approach for mega event transportation: integrating harmony search and black widow optimization

**DOI:** 10.7717/peerj-cs.2526

**Published:** 2024-12-10

**Authors:** Mohd Khaled Shambour, Esam Khan, Muhannad A. Abu-Hashem

**Affiliations:** 1 Department of Intelligent Systems Engineering, Faculty of Engineering and Design, Middle East University, Amman, Jordan; 2 Department of Information and Scientific Services, The Custodian of the Two Holy Mosques Institute for Hajj and Umrah Research, Umm Al-Qura University, Makkah, Saudi Arabia; 3 Department of Geomatics, Architecture and Planning, Faculty, King Abdul Aziz University, Jeddah, Saudi Arabia

**Keywords:** Optimization techniques, Transportation, Hajj Pilgrimage, Harmony search algorithm, Black widow optimization, Mega events

## Abstract

Optimization techniques have been extensively employed to address various challenges in human life across numerous domains. This study introduces a novel hybrid optimization algorithm that combines the strengths of harmony search (HS) and black widow optimization (BWO). The primary contribution lies in combining the cannibalism mechanism of BWO into the improvisation process of HS, enhancing HS’s ability to explore and refine solutions within the search space. The proposed Harmony Search Black Widow Optimization (HSBWO) algorithm is adapted and applied to a real-world optimization problem in transportation scheduling during the Hajj pilgrimage, particularly focusing on increasing the capacity of pilgrims’ housing sites in the Muzdalifah area by reusing the sites multiple times. Efficiently relocating a vast number of pilgrims to housing sites within a limited timeframe while meeting several hard and soft constraints is critical. Experimental results demonstrate that HSBWO consistently achieved the highest average scores across all tested scenarios compared to HS and BWO, with significant improvements in both solution quality and convergence rates. Statistical analysis using ANOVA confirmed that the performance differences were statistically significant at *α* equal to 0.05. Specifically, HSBWO outperformed HS with improvements in average fitness values ranging from 3.1% to 55.2%, while improvements over the BWO algorithm ranged from 6.4% to 56.0%, depending on the applied scenarios and population sizes.

## Introduction

The desire to meet human needs is constantly rising in today’s environment of rapid change due to technological breakthroughs and shifting preferences. In various domains of human life, optimization techniques have been widely employed to address a multitude of challenges.

The Harmony Search (HS) algorithm, introduced by Geem, Kim & Loganathan (2001), is one of the widely recognized evolutionary algorithms used for solving a diverse range of optimization problems (Qin, Zain & Zhou, 2022). Although the HS algorithm has effectively addressed various optimization challenges, it is susceptible to premature convergence and low optimization accuracy (Qin, Zain & Zhou, 2022). This limitation can result in suboptimal solutions, as the algorithm may get trapped in a local optimum.

The Hajj pilgrimage involves millions of people traveling to several holy sites in a limited period. The Hajj pilgrimage has unique logistical challenges, particularly with regard to accommodation and transportation issues. Among the critical bottlenecks is the Muzdalifah area, where pilgrims must spend a portion of the night before proceeding with their pilgrimage activities (Khan & Shambour, 2023). The limited capacity of housing sites in Muzdalifah necessitates efficient scheduling and resource utilization to accommodate the large number of pilgrims.

The motivation behind the research is rooted in addressing the inefficiencies of the HS algorithm, particularly in complex real-world problems like the transportation scheduling of pilgrims during Hajj. The shortcomings of relying solely on the HS algorithm, especially in terms of local search capabilities, underscore the necessity for a more robust and adaptive approach.

The central research question of this study is: How can the improvisation process of the HS algorithm be enhanced and adapted to optimize the scheduling process of transportation programs during large-scale events, accommodating individual preferences while improving overall transportation efficiency?

The development of an innovative method that can produce optimal solutions within time and resource restrictions is essential in addressing the challenges of managing transportation during the Hajj pilgrimage, given the different preferences of pilgrims. To solve this research challenge, a hybrid approach is developed integrating the HS algorithm and black widow optimization (BWO).

The main contribution of this article is the development of a hybrid approach that combines the HS algorithm with BWO (Hayyolalam & Pourhaji Kazem, 2020), called the Harmony Search Black Widow Optimization (HSBWO) algorithm. This hybrid algorithm significantly enhances the local search capability of the traditional HS algorithm by leveraging the cannibalism mechanism of BWO within the improvisation procedure of HS. This hybrid approach is designed to address the complex and large-scale transportation scheduling challenges of Hajj pilgrimage, where the HSBWO algorithm optimizes resource utilization by integrating the strengths of both optimization techniques.

This study’s importance is amplified by its focus on transportation optimization, a critical aspect of managing large-scale events. By developing a hybrid method that incorporates individual preferences, the research aims to enhance overall transportation efficiency during the Hajj pilgrimage. The insights gained from this study have the potential to benefit legislators, transit planners, and event organizers by offering innovative solutions for improving mobility and the overall experience of participants. Furthermore, the study’s findings contribute to the broader field of transportation optimization and can be applied to the management of mega-events. The article is structured as follows: ‘Problem Description’ provides a problem description, while ‘Related Literature’ reviews relevant literature. Section ‘Approach Implementation Framework’ outlines the approach implementation framework. The experimental results and discussion are presented in ‘Experimental Results and Discussion’, and ‘Conclusion’ concludes the article.

## Problem Description

This section offers comprehensive details regarding transportation programs, outlining the constraints utilized to regulate the scheduling process. Additionally, it presents the designed objective function, which serves as a guiding principle in the optimization process.

### Designed transportation programs

The study by Khan & Shambour (2023) examined the transportation process for a group of pilgrims traveling to Muzdalifah. They found that the standard unit of time for this process was 90 min. The total duration for assigning all the pilgrims to the Muzdalifah area was divided into seven time slots:

• Three time slots before midnight.
• Three time slots after midnight.
• One time slot after dawn.

This segmentation of the transportation period into different time slots helps facilitate the efficient management and scheduling of pilgrim movement to Muzdalifah. It allows for better coordination and a more organized, systematic assignment process.

Based on the arrival and departure timings at Muzdalifah, the researchers identified five main transportation programs:

• Main Program 1: Pilgrims arrive before midnight and depart before midnight.
• Main Program 2: Pilgrims arrive before midnight and depart after midnight.
• Main Program 3: Pilgrims arrive before midnight and depart after dawn.
• Main Program 4: Pilgrims arrive after midnight and depart after midnight.
• Main Program 5: Pilgrims arrive after midnight and depart after dawn.

From these five main programs, a total of 27 sub-programs were derived based on the distribution of pilgrims across different time slots. Table 1 delineates all sub-programs corresponding to each main program. Specifically, six sub-programs were derived from Main Program 1, nine sub-programs from Main Program 2, three sub-programs from Main Program 3, six sub-programs from Main Program 4, and three sub-programs from Main Program 5. These sub-programs are designed to streamline the scheduling process for pilgrims according to their specific arrival and departure time slots.

**Table 1:** Scheduling of all main and sub-program over seven time periods.

	Sub-program	Before midnight			After midnight			After Dawn
		TimeSlot 1	TimeSlot 2	TimeSlot 3	TimeSlot 4	TimeSlot 5	TimeSlot 6	TimeSlot 7
Main Program 1	SP1	**X**						
	SP2	**X**	**X**					
	SP3	**X**	**X**	**X**				
	SP4		**X**					
	SP5		**X**	**X**				
	SP6			**X**				
Main Program 2	SP7	**X**	**X**	**X**	**X**			
	SP8	**X**	**X**	**X**	**X**	**X**		
	SP9	**X**	**X**	**X**	**X**	**X**	**X**	
	SP10		**X**	**X**	**X**			
	SP11		**X**	**X**	**X**	**X**		
	SP12		**X**	**X**	**X**	**X**	**X**	
	SP13			**X**	**X**			
	SP14			**X**	**X**	**X**		
	SP15			**X**	**X**	**X**	**X**	
Main Program 3	SP16	**X**	**X**	**X**	**X**	**X**	**X**	**X**
	SP17		**X**	**X**	**X**	**X**	**X**	**X**
	SP18			**X**	**X**	**X**	**X**	**X**
Main Program 4	SP19				**X**			
	SP20				**X**	**X**		
	SP21				**X**	**X**	**X**	
	SP22					**X**		
	SP23					**X**	**X**	
	SP24						**X**	
Main Program 5	SP25				**X**	**X**	**X**	**X**
	SP26					**X**	**X**	**X**
	SP27						**X**	**X**

**Notes.**

X refers to a reserved slot.

### Problem formulation

To ensure the effectiveness of the proposed algorithm in achieving its objectives of providing diverse transportation programs to a large number of pilgrims, several constraints were implemented to guide its functionality. These constraints were designed to assist the algorithm in generating programs that offer a wide range of options, enabling pilgrims to select programs that best suit their individual preferences. This approach promotes inclusivity and enhances the overall satisfaction of pilgrims participating in the Hajj event.

There are two forms of constraints that control the program scheduling process: hard constraints, which must be satisfied in the final solution, and soft constraints, which allow for some relaxation or violation.

The problem is encoded using an assignment function (A) that maps various resources—such as pilgrim groups (PG), timeslots (T), Muzdalifah sites (S), and pilgrimage programs (P)—to their respective constraints. The representation scheme captures the relationships between these resources, ensuring that the constraints (both hard and soft) are respected during the solution generation process. The constraints are outlined as follows (Khan & Shambour, 2023):

Hard constraints:

H1. Each group of pilgrims is transported once. This is critical for ensuring fairness and avoiding any group being omitted or duplicated. The formula Eq. (1) expresses that for every pilgrim group *PG*_*j*_ , there is a unique assignment A to a Muzdalifah site *S*_*i*_, meaning no pilgrim group is left unassigned or assigned more than once.

(1) \begin{eqnarray*}{A}_{PG}^{S}={A}_{P{G}_{j}}^{{S}_{i}} \forall j\in PG;i\in S\end{eqnarray*}

H2. Each timeslot contains only one pilgrim group, avoiding any conflicts or overlaps. This constraint is designed to prevent conflicts and overcrowding by ensuring that no two pilgrim groups are assigned to the same timeslot and site simultaneously. Equation (2) mathematically represents this by ensuring that for any timeslot *T*_*t*_ and site *S*_*i*_, the assignment of pilgrim group *PG*_*j*_ does not overlap with another group *PG*_*k*_.

(2) \begin{eqnarray*}{A}_{P{G}_{j}}^{{T}_{t},{S}_{i}}\not = {A}_{P{G}_{k}}^{{T}_{t},{S}_{i}}t\in T;j\not = k; \forall j,k\in PG;i\in S\end{eqnarray*}

H3. The generated solutions must contain all main transportation programs. To ensure diversity in the transportation programs, this constraint enforces that the final scheduling solution contains a minimum percentage of each main program. Equation (3) demonstrates that each pilgrim group *PG*_*j*_ is assigned to a program *P*_*i*_, with the assignment percentages meeting the minimum threshold *x*_*i*_ for each program *i*. The specified values *x*_*i*_ ensure that no program is underrepresented.

(3) \begin{eqnarray*}{A}_{PG}^{P}={A}_{P{G}_{j}}^{{P}_{i}} \forall i\in P;j\in PG\end{eqnarray*}

such that $X \left( {A}_{PG}^{{P}_{i}} \right) \geq {x}_{i}\forall i\in P$, where *X* indicates the assignment percentages of the main pilgrim groups. The *x*_*i*_ represents the percentage of the main program *i*, such that *x*_*i*=1_ =*0.02, x*_*i*=2_ =0.03, *x*_*i*=3_ =0.05, *x*_*i*=4_ =0.01, and *x*_*i*=5_ =0.01.

Soft constraints:

S1. Pilgrims should be distributed among the main programs based on the preferred percentage for each main program. This soft constraint aims to align the distribution of pilgrims with preferred percentages for each program. Equation (4) expresses this by ensuring that the assignment percentage function *Y* for each program *P*_*i*_ closely matches the desired proportion *y*_*i*_. While this constraint allows for some deviation, the goal is to approach the target distribution as closely as possible.

(4) \begin{eqnarray*}Y \left( {A}_{PG}^{{P}_{i}} \right) \cong {y}_{i} \forall i\in P\end{eqnarray*}

such that *y*_*i*=1_ = 0.2, *y*_*i*=2_ = 0.5, *y*_*i*=3_ = 0.2, *y*_*i*=4_ = 0.01,  *and y*_*i*=5_ = 0.09.

S2. All timeslots are occupied by pilgrim groups across all Muzdalifah sites. This constraint ensures that all available timeslots and sites are utilized, avoiding any unused resources. Equation (5) states that for each timeslot *T*_*t*_ and site *S*_*i*_ , there must be at least one assigned pilgrim group *PG*_*j*_ . This helps maximize the use of all available timeslots and locations, promoting efficiency.

(5) \begin{eqnarray*}{A}_{P{G}_{j}}^{{T}_{t,}{S}_{i}}\not = \varnothing  \forall t\in T; \forall i\in S;j\in PG\end{eqnarray*}

S3. Utilize each Muzdalifah site as much as possible by allocating the largest possible number of pilgrim groups. This soft constraint encourages the allocation of as many pilgrim groups as possible to each site. Equation (6) represents this by aiming to maximize the number of pilgrim groups *nPG* assigned to a site *S*_*i*_. While some flexibility is allowed, the objective is to use each site to its full capacity.

(6) \begin{eqnarray*}{A}_{PG}^{{S}_{i}}\cong Max \left( nPG \right)  \forall i\in S\end{eqnarray*}

The minimization objective function serves as a metric to assess the quality of the generated solutions and guides in determining the most suitable distribution for transporting pilgrim groups to Muzdalifah sites. This objective function assigns a numerical value to each solution, reflecting the efficiency of the final solution. Since the objective of this study is to achieve the optimal distribution of transportation programs while adhering to as many constraints as possible, the costs associated with violating these constraints are determined based on their significance in attaining the final solution. The objective function, which accounts for the costs associated with violating both hard and soft constraints, is represented as illustrated in Eqs. (7)–(13):

(7) \begin{eqnarray*}Objective~Functio~nCost=Cost \left( Hard~Constraints~Violations \right) \nonumber\\\displaystyle +Cost \left( Soft~Constraints~Violations \right) \end{eqnarray*}

(8) \begin{eqnarray*}Cost \left( Hard~Constraints~Violations \right) =1,000\times (Number~of~violated~hard~constraints)\end{eqnarray*}

(9) \begin{eqnarray*}Cost(Soft~Constraints~Violations)=Cost(S1~Violations)+Cost(S2~Violations)\nonumber\\\displaystyle +Cost(S3~Violations).\end{eqnarray*}

Where:

(10) \begin{eqnarray*}Cost(S1~Violations)=10\times (abs(Actual~distribution~percentage~for~each~main~program-\nonumber\\\displaystyle Preferred~distribution~percentage~for~each~main~program))\end{eqnarray*}

(11) \begin{eqnarray*}Cost(S2Violations)=5\times (Number~of~unoccupied~time~slots)\end{eqnarray*}

(12) \begin{eqnarray*}Cost \left( S3~Violations \right) =Number~of~times~lots~without~a~new~sub\text{-}program~assignment.\end{eqnarray*}

The fitness function in this problem is defined as a minimization objective function that measures the quality of the solution. The function assigns costs to violations of both hard and soft constraints, with hard constraints carrying a higher penalty (*e.g.*, a multiple of 1,000 per violation). Soft constraints are penalized based on the degree of deviation from the desired distribution or usage, with different weights applied (*e.g.*, 10 for distribution discrepancies, 5 for unoccupied time slots). The fitness function guides the algorithm toward minimizing these costs and thus improving the quality of the solution. Accordingly, the objective function is represented as follows:

(13) \begin{eqnarray*}Min\sum _{h=1}^{3}{n}_{{v}_{{H}_{h}}}\times {W}_{{H}_{h}}+\sum _{s=1}^{3}{n}_{{v}_{{S}_{s}}}\times {W}_{{S}_{s}}\end{eqnarray*}

where *n*_*v*_*H*_*h*___ represents the violation times for each of the hard constraints (*H*_1_, *H*_2_, *H*_3_), *n*_*v*_*S*_*s*___ denotes the violation times for each of the soft constraints $ \left( {S}_{1},{S}_{2},{S}_{3} \right) ,$ *W*_*H*_*h*__ and *W*_*S*_*s*__ indicate the violation cost values for the hard and soft constraints, respectively.

## Related Literature

In recent years, significant research efforts have been directed toward tackling the challenges and improving various aspects of mega-event transportation and management. Scholars and specialists have researched several ways and techniques to improve participants’ overall experience, satisfaction, and efficiency during large-scale events. In this section, we present an overview of the relevant literature that has contributed to the optimization of transportation and accommodation for large-scale events, with a particular emphasis on the Hajj pilgrimage. The investigations covered a variety of technologies, including artificial intelligence, optimization algorithms, simulation modeling, and scheduling strategies. By reviewing past publications, we gain useful insights into existing methodologies and identify the shortcomings that our proposed hybrid scheduling approach seeks to resolve.

Shambour, Khan & Salibi (2017) created a framework for the effective distribution of Mina campgrounds, specifically designed to accommodate more than two million pilgrims. The motivation behind their work is because of Mina’s small size, which poses a major obstacle to the best arrangement of pilgrims’ camps and amenities. The proposed framework is based on the application of artificial intelligence techniques using geographical data for the entire Mina region, considering soft and inelastic constraints. The recommendations indicated that the proposed solutions help to benefit from the maximum capacity of the available resources, which will lead to an increase in the capacity of the Mina shrine. In another work, Shambour & Khan (2019) suggested a method for assigning pilgrims to Mina camps. The proposed algorithm proved to be successful in providing space for pilgrims, as 80% of the pilgrims were given an area of 76.2% of the total accommodation available in the Mina region.

Focusing on security and time preferences during the optimization process, Rehman & Felemban (2019) proposed an interactive scheduling method for groups of pilgrims in which groups of pilgrims can be scheduled according to their preferences when performing stoning rituals at the Jamarat Building. The results obtained when the pilgrims’ journeys were rescheduled using this strategy during the Hajj season in 1440 AH proved good.

Felemban et al. (2017) used cameras within the Grand Mosque in Makkah to simulate crowd movements around the Kaaba. Mass-Motion software was used to develop their model, which was crafted in large part using data gathered from these cameras. The results of the model showed important information about the density and movement patterns of the crowds, especially in the important areas of the Great Mosque of Makkah, which helps to maintain the safety of the crowds and saves effort and cost in the process of organizing the crowds.

Morgan & Khayyat (2022) proposed an improved approach to shortening the distance between service places in the holy sites. The researchers used the genetic algorithm (GA) to achieve the best distribution of service points in the holy sites for visitors and pilgrims to obtain maximum performance. Points of service include police cars, ambulances, fire engines, food trucks, water and other beverages, and security cameras. As a case study, the two researchers applied an algorithm to distribute ambulances in the crowded holy Arafat area.

Al-Sabban & Ramadan (2005) developed a model to simulate the shuttle bus system. For loop 1 (Al-Nafrah), the simulation suggests maintaining a minimum of around 500 buses to ensure a tolerable Arafat evacuation time. Short inter-dispatch periods between bus caravans should be avoided to prevent lengthening the journey time from Arafat to Muzdalifah. For loop 2 (from Muzdalifah to Mina), the optimal number of main shuttle buses is between 231 and 297 to reduce the journey time from Muzdalifah to Mina without extending the overall Muzdalifah evacuation duration. It’s recommended to assign two drivers per bus on a time-shift basis to handle the increased number of rounds per bus when reducing the number of shuttle buses. Large inter-dispatch delays between bus caravans should also be avoided to prevent lengthening the journey time from Muzdalifah to Mina. A study aimed at enhancing the operational planning of the shuttle bus service within the Hajj Establishment was proposed by Hussain, Felemban & Ur Rehman (2021). The goal is to improve performance by determining the optimal number of buses and cycles needed for each office in the establishment.

Haase et al. (2019) introduced a model and a solution approach to optimize the scheduling of pilgrims performing the stoning ritual. The pilgrim schedule prescribes specific routes and time slots for all registered pilgrim groups. According to the numerical analysis, the method often resolves cases with over 2.3 million variables in less than ten minutes. Simultaneously, the difference between the upper bound and the optimal solution never goes over 0.28%. Yasein & Khan (2023) constructed a computer simulation of the Mashaaer Holy Site’s shuttle bus system for pilgrims. Using a multimodal modeling and simulation tool created by AnyLogic, the model was created to incorporate activities that took place during the pilgrims’ shuttle bus transportation from Arafat to Muzdalifa. The model may be used to optimize the pilgrim’s transportation system in terms of several parameters by determining how effective the present system is and then making recommendations for improvements that could improve performance.

In another study, Felemban, Fatani & Rehman (2019) presented an optimized scheduling process that schedules the movement of pilgrims using trains to perform spatiotemporal rituals safely. The scheduling process considered several factors: the type of train movement (D for Muzdalifah to Mina, C for Arafat to Muzdalifah, or B for Mina to Arafat), the camping locations of pilgrims, the camping destinations of other pilgrims, and the permitted roadways to and from camps and stations. Additionally, the scheduling procedure considers station capacity to prevent overcrowding incidents.

Furthermore, Liao et al. (2023) proposed a strategy for strategically placing fences to direct traffic and ease congestion in public areas. A congestion probability social force model (CP-SFM) is introduced by the authors in order to assess various fence design and simulate pedestrian behavior. In order to optimize the fence arrangement, they frame the issue as one of optimization and suggest the ant colony crowd intervention method (ACCI). Finally, the authors show how well ACCI works to prevent crowding in a variety of settings, including two actual subway stations.

Additionally, Elkhouly, Tamaki & Iwasaki (2023) offered two methods for crowd management at major sporting events in order to relieve traffic at surrounding transit hubs. The first strategy delays fans’ arrival at the closest station and promotes walking to a different nearby station by establishing fan attraction locations using augmented reality and wearable technology. The second method increases the number of fans who leave crowded stations by using wearable technology and a smartphone application to give wayfinding and intra-station navigation for fans traveling in groups. Additionally, the authors offer agent-based simulations to show how effective these methods are at reducing station congestion.

In addition, Lu et al. (2023) introduced a technique for precise real-time forecasting of the intense passenger inflow (IPF) brought on by special events at urban rail transit (URT) stations. The system models the association between historical special events and passenger characteristics by combining individual travel card data, event data, station data, and other information. The IPF is estimated using the system’s online inflow prediction and offline model training components, which significantly reduce estimation error as compared to conventional prediction models.

Huang et al. (2020) introduced a novel approach to solve the evacuation route optimization (EPO) issue in crowd and catastrophe management called the ant colony evacuation planner (ACEP). The joint finding of a set of ideal evacuation routes is made possible by the ACEP, which simulates crowd behavior during evacuation using the entire colony of ants. To speed up and enhance the efficiency of ACEP, the authors also present the incremental flow assignment (IFA) technique. The ACEP technique shows good results when tested numerically on networks of various sizes.

Recently, Khan & Shambour (2023) studied the issue of accommodating the greatest number of pilgrims at Muzdalifah locations while they were on pilgrimage using HS algorithm. The research aims to accommodate more than one group of pilgrims at each site in Muzdalifah instead of a single pilgrim group, as in the current status. The outcomes demonstrated that it would be possible to triple the capacity of Muzdalifah’s accommodation sites compared to the current situation. However, despite the effectiveness of HS algorithm in improving the accommodation capacity, the HS algorithm exhibited limitations, particularly in its local search capabilities, which restricted its ability to fully optimize the complex scheduling problem. This limitation highlighted the need for further enhancements to achieve better solutions, especially in the context of large-scale event management.

The studies presented in this section offer insight into various aspects of transportation and accommodation optimization for large-scale events, particularly the Hajj. To address the complicated challenges of large-scale event management, these studies used a variety of methodologies and techniques, such as optimization algorithms, simulation modeling, and scheduling systems.

While previous studies have made significant contributions to various aspects of transportation in mega-events, there is still a gap in optimizing the complex scheduling challenges to transport large groups of pilgrims with diverse preferences. Specifically, although the HS algorithm has been applied in related works, it suffers from limitations in local search capabilities and tends to converge prematurely. This article addresses these gaps by presenting a hybrid optimization approach that combines HS and BWO, leveraging the strengths of both algorithms to enhance local search and global exploration. This combination aims to provide a more robust and efficient solution to the complex scheduling issues in real-world problems.

## Approach Implementation Framework

This section briefly describes the HS and BWO algorithms, outlining their operators and characteristics. Additionally, it provides a detailed description of the proposed approach employed in this study.

### Harmony Search algorithm

The HS algorithm is an evolutionary algorithm inspired by the principles of biological evolution found in nature, as proposed by Geem, Kim & Loganathan (2001). Its mechanism involves maintaining a set of solutions throughout the search process, where these solutions collaborate to generate a new solution in each iteration.

• HS algorithm operators are (Geem, Kim & Loganathan, 2001; Shambour et al., 2014):
  – Harmony initialization: Producing a starting population of harmonies at random.
  – Harmony memory consideration: Determining the procedure for constructing a new harmony from either the HM or through random search.
  – Pitch adjustment: Adjusting the content or values of specific elements in a new harmony.
  – Harmony memory update: Updating the contents of the HM by including the best harmonies in the HM according to their fitness values.
• Characteristics of the HS algorithm (Alia & Mandava, 2011; Shambour, 2018):
  – Ease of implementation.
  – HS uses a stochastic search technique influenced by music improvisation.
  – Pitch modification and harmony memory consideration are two ways the algorithm strikes a balance between exploration and exploitation.
  – HS is capable of handling both discrete and continuous optimization problems and does not require gradient information.
  – It has been effective in solving a variety of optimization issues.

### Black Widow Optimization algorithm

The BWO algorithm is an evolutionary algorithm inspired by the real-life mating process of black widow spiders in nature, as proposed by Hayyolalam & Pourhaji Kazem (2020). Spiders share information, including their positions or attributes, to facilitate collaboration and information exchange. This collaborative effort enables spiders to collectively generate a new solution or update their positions for subsequent iterations.

• The BWO algorithm’s operators are Hayyolalam & Pourhaji Kazem (2020):
  – Population initialization: Generating an initial population of potential solutions randomly, often referred to as “spiders.”
  – Procreation: determining which spiders will be selected for the next round of reproduction or survival in the subsequent generation process.
  – Cannibalism: eliminating fewer fit spiders and promoting the survival of stronger ones.
  – Mutation: introducing small stochastic changes to the genetic makeup of spiders within the population.
• Characteristics of the BWO algorithm (Abu-Hashem & Shambour, 2024; Madhiarasan, Cotfas & Cotfas, 2023):
  – Ease of implementation.
  – Efficiently navigate the search space, allowing for quicker identification of optimal or near-optimal solutions.
  – Ability to avoid local optima by circumventing the problem of getting trapped in local optima.
  – Exhibiting satisfactory accuracy in finding solutions.
  – Reduced complexity.

### Proposed HSBWO approach

The proposed approach in this study is a modification of the algorithm presented by Khan & Shambour (2023). This new approach combines the HS algorithm and the BWO algorithm to create a hybrid approach called HSBWO. The primary goal of this hybrid approach is to optimize the scheduling procedures for transportation programs in the Muzdalifah area. By leveraging the strengths of both algorithms, HSBWO aims to deliver a complete solution that accurately represents the scheduled assignment programs for all locations in the Muzdalifah area.

### Step 1. Initializing the algorithm and problem parameters

The initialization step involves determining the parameters for the scheduling programs. These parameters include the number of locations, main programs, sub-programs, distribution ratios, timeslots, and other relevant factors. Additionally, specific parameters related to the algorithm itself are initialized. The HSBWO algorithm parameters comprise the following:

• Harmony memory size: refers to the number of solutions stored in the memory of the HSBWO algorithm.
• Harmony memory consideration rate (HMCR): HMCR is a parameter that determines the balance between adopting solutions from HM or through random search. Where 0 ≤ *HMCR* ≤ 1.
• Pitch adjustment rate (PAR): The PAR is a parameter used to facilitate the modification of previously chosen pilgrim groups from the assigned programs stored in the HM. It provides the possibility of generating new harmony that is adjacent to the previous solutions, thereby intensifying the search in the vicinity of the current solutions. The pitch adjustment operator is defined as follows:

\begin{eqnarray*}PAR= \left\{ \begin{array}{@{}l@{}} \displaystyle P1 0\leq U(0,1)< 0.33 \\ \displaystyle P2 0.33\leq U(0,1)< 0.66 \\ \displaystyle P3 0.66\leq U(0,1)\leq 1  \end{array} \right. \end{eqnarray*}

where $U \left( 0,1 \right) $ represents a uniform random distribution ranging between 0 and 1. The procedures P1, P2, and P3 are detailed as follows:

• *P1*: Choosing two random timeslots from the currently scheduled programs and identifying the subprograms associated with the chosen timeslots.
• *P2*: Removing the selected subprograms from the current schedule and making their timeslots empty.
• *P3*: Developing one or more subprograms that cover empty timeslots.

### Step 2. Initialization of HM

During the initialization of the HM, the algorithm generates initial solutions (denoted as H) for assigned programs using the greedy search technique. These solutions are then stored in HM as the starting set of solutions as shown below:

\begin{eqnarray*}HM= \left[ \begin{array}{@{}cccc@{}} \displaystyle {h}_{1}^{1}&\displaystyle {h}_{2}^{1}&\displaystyle .....&\displaystyle {h}_{N}^{1}\\ \displaystyle {h}_{1}^{2}&\displaystyle {h}_{2}^{2}&\displaystyle .....&\displaystyle {h}_{N}^{2}\\ \displaystyle \vdots &\displaystyle \vdots &\displaystyle \vdots &\displaystyle \vdots \\ \displaystyle {h}_{1}^{HMS}&\displaystyle {h}_{2}^{HMS}&\displaystyle .....&\displaystyle {h}_{N}^{HMS} \end{array} \right] \left[ \begin{array}{@{}c@{}} \displaystyle f({H}^{1})\\ \displaystyle f({H}^{2})\\ \displaystyle \vdots \\ \displaystyle f({H}^{HMS}) \end{array} \right] \end{eqnarray*}

### Step 3. Improvisation process

The improvisation process comprises the following stages:

**Stage 1.** Applying the conventional procedure of the HS algorithm to generate a New Harmony (NH) by utilizing the HS algorithm operators (*i.e.,* HMC and PA).

**Stage 2.** Applying the cannibalism operation of the BWO algorithm that relies on eating members of the same species. The following are the steps of the cannibalism process in detail:

• Selection of harmonies: In this step, two solutions (harmonies) are randomly selected from the population. These harmonies are labeled as *H*_*Y*_ and *H*_*Z*_.
• Swap mutation process: A swap mutation is performed between the selected harmonies where some components of these two harmonies are exchanged. The number of times this swap occurs depends on a predefined parameter called the number of cannibalism (*nCann)*.
• Generation of new harmonies: After performing the swap process, two new harmonies *H*_*Y*′_ and *H*_*Z*′_ are created. This process introduces variability into the population and helps prevent the algorithm from getting stuck in local optima.

Mathematically, the swap mutation between *H*_*Y*_ and *H*_*Z*_ for a specified *nCann*, is represented as follows:

\begin{eqnarray*}[{H}_{Y}^{{^{\prime}}},{H}_{Z}^{{^{\prime}}}]=Mutate({H}_{Y},{H}_{Z},nCann) \end{eqnarray*}

This swapping procedure of harmony memory (HM) components aims to maintain diversity among harmonies and reduce the likelihood of premature convergence by introducing differences into the solution space. The proposed combination of the HS and BWO algorithms achieves a balance between intensification and diversification in the search process, resulting in more robust optimization performance.

### Step 4. Update HM

Newly created harmonies (NHs) undergo an evaluation process using an objective function (*F*). Only harmonies with good qualities will be kept in the HM. The updated rule is given as follows:

\begin{eqnarray*}HM=best([HM,F(HM)]\cup [NHs,F(NHs)]) \end{eqnarray*}

where *best* selects the harmonies with the highest fitness values.

### Step 5. Stopping Criterion

**Figure 1:**
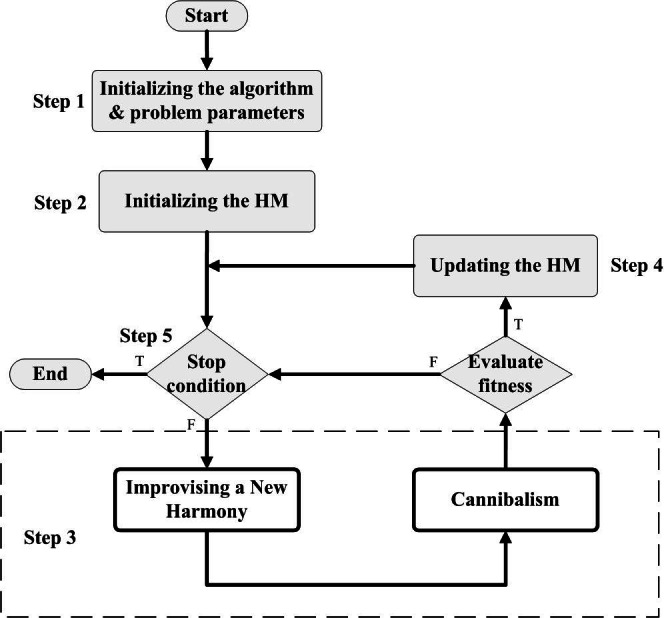
The basic flowchart of the proposed HSBWO algorithm.

Indicating whether to continue or end the improvisation cycle based on a predetermined condition, such as the number of evaluations (NE). The basic flowchart of the proposed HSBWO algorithm is given in Fig. 1, and the pseudo-code algorithm is provided in Algorithm 1 .

**Table:** 

Algorithm 1. The pseudocode of the proposed HSBWO algorithm
**Step 1:** **Set the algorithm and problem parameters**
Including *NE*, *HMS*, *HM*, *HMCR, PAR*, *NoPrograms*, *NumOfLocations*, *NH*, *pCan*, *nCann*, $MinProgRate= \left[ 0.02,0.03,0.05,0.01,0.01 \right] ,DesiredProgRate= \left[ 0.2,0.5,0.2,0.01,0.09 \right] $
**Step 2: Initialize HM with a number of solutions according to HMS**
**For***i* = 1 to HMS
*H*^*i*^=*GenerateIntialSol* () // *Initialize* *HM*
*F*(*H*^*i*^) = *f*(*H*^*i*^)// *Find* *objective* *function* *cost*
*HM*^*i*^ = [*H*^*i*^, *F*(*H*^*i*^)]
**End For**
**Step 3: Improvisation Process**
*NH* = []// *Initialize* *a* *New* *Harmony* (*NH*)[*NewHarmonies*]// *Initialize* *a* *New* *Harmonies* *Pool*
**For** i =1 to *NI*// *NI* *is* *the* *number* *of* *improvization*
*H*^*X*^ = *Select* *X* (HM) //$~~Select~a~random~harmony~{H}^{x}~wherex\in \left[ 1,HMS \right] $
**For** j =1 to Num Of Locations
**IF**$ \left( rnd\leq HMCR \right) $ // ***Memory Consideration***
*NH*^*j*^ = *H*^*X*,*j*^
**IF**$ \left( rnd\leq PAR \right) $ // ***Pitch Adjustment***
$ \left[ StartSlot,~EndtSlot \right] =return~Start~EndSlots//$ *Select two random slots from location j of a NH*
$N{H}^{j, \left[ StratSlot,EndSlot \right] }=Assign~Rnd~Prog \left( StratSlot,EndSlot \right) //~~Assign~random~Program(s)~within$*the* *StratSlot* *and* *EndSlot*
**End IF**
**Else**
$N{H}^{j}=Assign~Rnd~Prog \left( ~ \right) //~~Assign~random~program \left( s \right) at~j~location~of~a~NH$
**End** **IF**
**End** **For**
$ \left[ NewHarmonies,F \left( NewHarmonies \right) \right] $
$= \left[ NewHarmonies,F \left( NewHarmonies \right) \right] \cup [NH,f \left( NH \right) ]//~~Add~a~NH~to~the~New~Harmonies~pool$
**For** *n* = 1 *to* n Cann// ***Cannibalism***
$[{H}^{Y},{H}^{Z}]=SelectYZ \left( HM \right) //~~Select~two~random~harmonies{H}^{y},{H}^{z}where~y,z\in \left[ 1,HMS \right] $
[*H*^*Y*,*p*^, *H*^*Z*,*q*^] = *Select* *Locations*(*H*^*Y*^, *H*^*Z*^)// *Select* *two* *random* *locations* *p* *and* *q* *fromH*^*Y*^, *H*^*Z*^*respectively*
[*H*′^*Y*^, *H*′^*Z*^] = *Mutate*(*H*^*Y*,*p*^, *H*^*Z*,*q*^)// *Perform* *Swap* *Mutation* *between* *p* *and* *q* *locations* *ofH*^*Y*^*andH*^*Z*^
$ \left[ NewHarmonies,F \left( NewHarmonies \right) \right] = \left[ NewHarmonies,F \left( NewHarmonies \right) \right] \cup \left( {{H}^{{^{\prime}}}}^{Y},f \left( {{H}^{{^{\prime}}}}^{Y} \right) \right) $
$ \left[ New~Harmonies,F \left( New~Harmonies \right) \right] = \left[ New~Harmonies,F(New~Harmonies) \right] \cup \left( {H}^{Z{^{\prime}}},f \left( {H}^{Z{^{\prime}}} \right) \right) $
**End** ** For**
Sort $ \left( NewHarmonies,F \left( NewHarmonies \right) \right) //~~Sort~New~Harmonies~pool~according~to~F \left( NewHarmonies \right) $
**End** ** For**
** Step 4: Update the HM**
$HM=bestof \left( \left[ HM,F \left( HM \right) \right] \cap [NewHarmonies,F \left( NewHarmonies \right) ] \right) //~~Keep~best~harmonies$*according* *to* *their* *fitness* *values*
**Step 5: Stopping improvisation if the termination criterion is met; otherwise go to Step 3**.

## Experimental Results and Discussion

In this section, the simulation results of applying the proposed HSBWO algorithm are presented, along with an analysis of the influence of the algorithm’s parameters on the obtained results. The case study focuses on scheduling pilgrim programs across one hundred sites, which remained constant throughout all experiments in this study. The proposed algorithm was evaluated through 30 trials, each involving 1,000 iterations of the improvisation cycle.

### Experimental design

As shown in Table 2, eight scenarios with various parameter settings were constructed for the comparison algorithm. Thirty trials were carried out for each scenario with different population sizes of 5, 20, 50, and 100. It is worth noting that the parameter setting for the BWO algorithm “cannibalism rate” was set to 0.44, as recommended in Hayyolalam & Pourhaji Kazem (2020). These experimental settings were chosen to examine the exploration and exploitation search efficacy compared to HS (Khan & Shambour, 2023), BWO (Hayyolalam & Pourhaji Kazem, 2020), and HSBWO algorithms. Several statistical measurements were employed in the comparison of the studied algorithms, including the mean, standard deviation, and best and worst fitness values. All experiments were coded using MATLAB 2020b, which was run on Windows 11 64-bit 12th Gen Intel(R) Core (TM) i7-12650H 2.30 GHz and 24 GB of RAM.

**Table 2:** Parameter settings of HS and BWO algorithms.

Algorithms		Sc.1	Sc.2	Sc.3	Sc.4	Sc.5	Sc.6	Sc.7	Sc.8
*HSBWO*	*HMCR Procreate rate*	0.3	0.3	0.5	0.5	0.7	0.7	0.9	0.9
*HS*	*PAR*	0.3	0.5	0.3	0.5	0.3	0.5	0.3	0.5
*BWO*	*Mutation rate*	0.7	–	0.5	–	0.3	–	0.1	–

### Experimental results

#### Simulation results with a population size of five

The experimental outcomes for a population size of five, as detailed in Table 3, reveal that the proposed HSBWO algorithm ( Algorithm 1 ) consistently outperforms other algorithms. It achieves the highest average fitness scores across all test scenarios (highlighted in bold), demonstrating its superior optimization capabilities. Notably, the HS algorithm also surpasses the performance of the BWO algorithm in all scenarios, reinforcing its effectiveness within the hybrid approach.

**Table 3:** Statistical results of fitness values for 30 experimental tests with a population size of five.

Scenarios		Sc1	Sc2	Sc3	Sc4	Sc5	Sc6	Sc7	Sc8
HSBWO	***Mean***	**1,108**	**1,092.3**	**1,133.8**	**1,248.6**	**1,255.2**	**1,326.3**	**1,689.6**	**2,037.6**
	***Std***	335.1	309.7	328.9	447.2	461.1	445.7	504.5	375.8
	***Best***	866	893	925	931	897	973	1,068	1,173
	***Worst***	1,995	2,065	1,971	2,063	2,095	2,136	2,198	2,268
HS	***Mean***	2,420.5	2,437.3	2,410.1	2,430.7	2,382.1	2,422.5	2,349.3	2,376.1
	***Std***	14.701	16.735	16.8	11.4	15.0	14.7	43.3	111.4
	***Best***	2,389	2,397	2,384	2,410	2,349	2,399	2,289	1,792
	***Worst***	2,445	2,465	2,441	2,449	2,407	2,454	2,553	2,430
BWO	***Mean***	2,517.6	–	2,646.9	–	2,581.8	–	2,706.2	–
	***Std***	25.8	–	37.3	–	41.9	–	31.7	–
	***Best***	2,467	–	2,589	–	2,488	–	2,646	–
	***Worst***	2,563	–	2,720	–	2,655	–	2,759	–

**Notes.**

The best mean results are highlighted in bold font.

**Figure 2:**
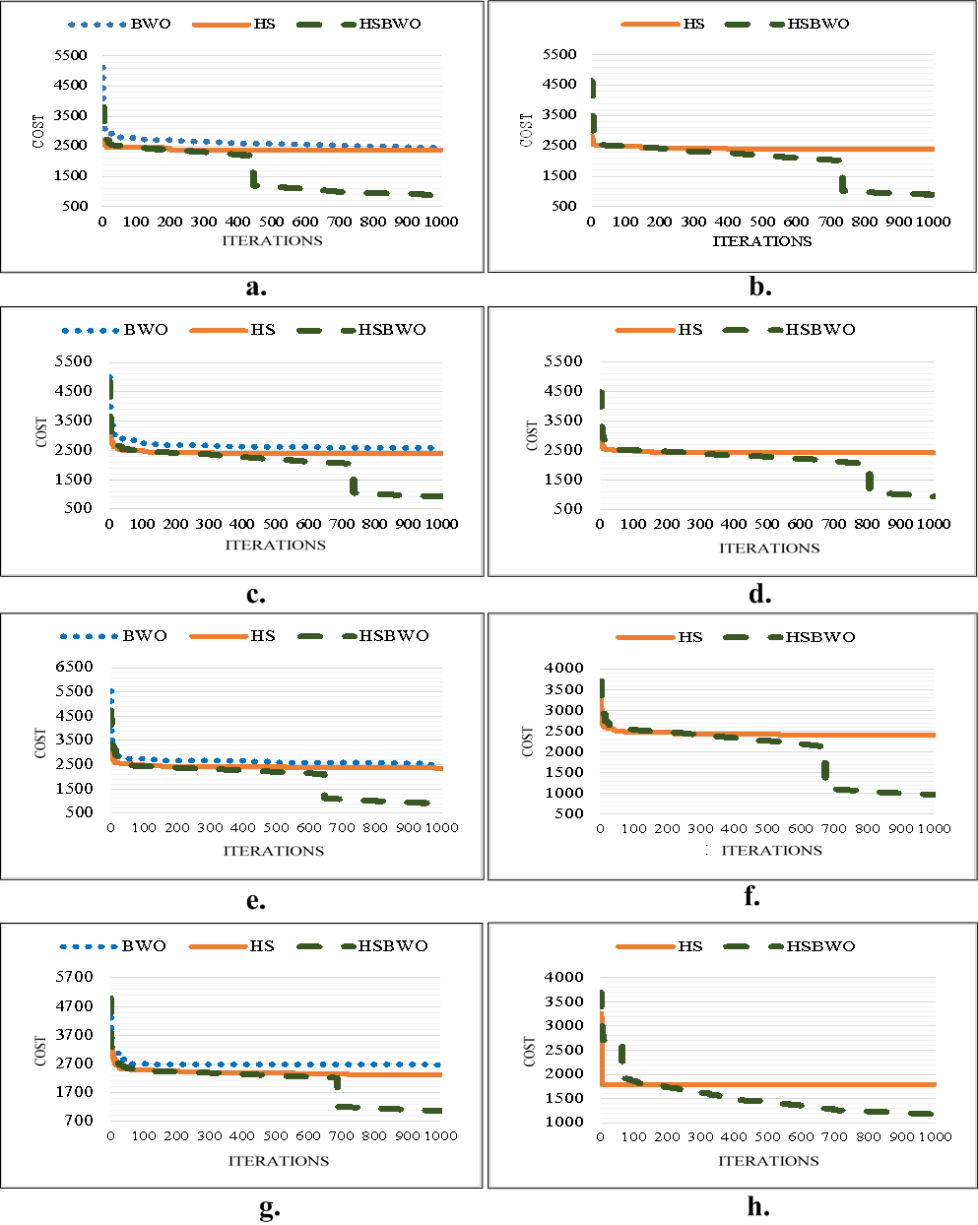
The comparison of convergence rates (population size of 5). (A) Scenario 1. (B) Scenario 2. (C) Scenario 3. (D) Scenario 4. (E) Scenario 5. (F) Scenario 6.

Figure 2 provides a visual comparison of the best convergence trends across various parameter settings for the algorithms. These results highlight the efficiency and reliability of the HSBWO in providing higher solution qualities.

To further validate these findings, an ANOVA statistical analysis was conducted. The goal was to determine if there were statistically significant differences between the fitness results of the compared algorithms. The hypotheses for the ANOVA test were as follows:

h0: The average fitness values of the compared algorithms are equal (*i.e., μ*1 = *μ*2 = *μ*3, where *μ* is the mean).

h1: At least one algorithm’s average fitness value is significantly different from the others.

The ANOVA results, shown in Table 4, indicate a significant difference in the mean fitness values, as the *P*-values are consistently lower than the 0.05 significance threshold. This statistical evidence leads to the rejection of the null hypothesis (h0) and supports the alternative hypothesis (h1), confirming that the fitness results differ significantly between the algorithms.

• Simulation results with a population size of 20

When the population size is increased to 20, the experimental results, as presented in Table 5, continue to demonstrate the superiority of the HSBWO algorithm. It achieves the highest average fitness scores in all scenarios (highlighted in bold), further solidifying its robustness and effectiveness. The HS algorithm also maintains its advantage over the BWO algorithm, consistently ranking second.

**Table 4:** ANOVA test results for the compared algorithms with a 5-population size.

Scenarios	*F*-Value	*P*-Value	*F*-Critical
Sc1	4.93E+02	3.50E−48	3.10E+00
Sc2	5.64E+02	1.43E−31	4.01E+00
Sc3	5.43E+02	7.35E−50	3.10E+00
Sc4	2.56E+02	3.37E−37	3.10E+00
Sc5	2.15E+02	2.32E−34	3.10E+00
Sc6	2.15E+02	2.06E−34	3.10E+00
Sc7	9.30E+01	2.47E−22	3.10E+00
Sc8	6.59E+01	3.72E−18	3.10E+00

**Table 5:** Statistical results of fitness values for 30 experimental tests with a population size of 20.

Scenarios		Sc1	Sc2	Sc3	Sc4	Sc5	Sc6	Sc7	Sc8
HSBWO	***Mean***	**2,074.9**	**2,136.2**	**2,146.6**	**2,064.0**	**2,122.8**	**2,133.6**	**1,910.9**	**2,183.3**
	***Std***	276.37	240.12	186.0	307.0	280.8	248.6	372.1	177.5
	***Best***	1,217	1,223	1,783	1,268	1,494	1,274	1,405	1,720
	***Worst***	2,336	2,337	2,353	2,358	2,337	2,379	2,387	2,428
HS	***Mean***	2,431.1	2,440	2,416.3	2,437.1	2,399.4	2,433.8	2,369.4	2,417.7
	***Std***	13.232	18.048	17.296	12.213	12.207	16.65	21.248	23.783
	***Best***	2,404	2,397	2,373	2,418	2,376	2,382	2,325	2,395
	***Worst***	2,454	2,468	2,454	2,460	2,424	2,457	2,427	2,488
BWO	***Mean***	2,679	–	2,660.1	–	2,656.5	–	2,658.3	–
	***Std***	31.3	–	30.847	–	28.717	–	26.361	–
	***Best***	2,620	–	2,565	–	2,591	–	2,592	–
	***Worst***	2,752	–	2,705	–	2,726	–	2,725	–

**Notes.**

The best mean results are highlighted in bold font.

**Figure 3:**
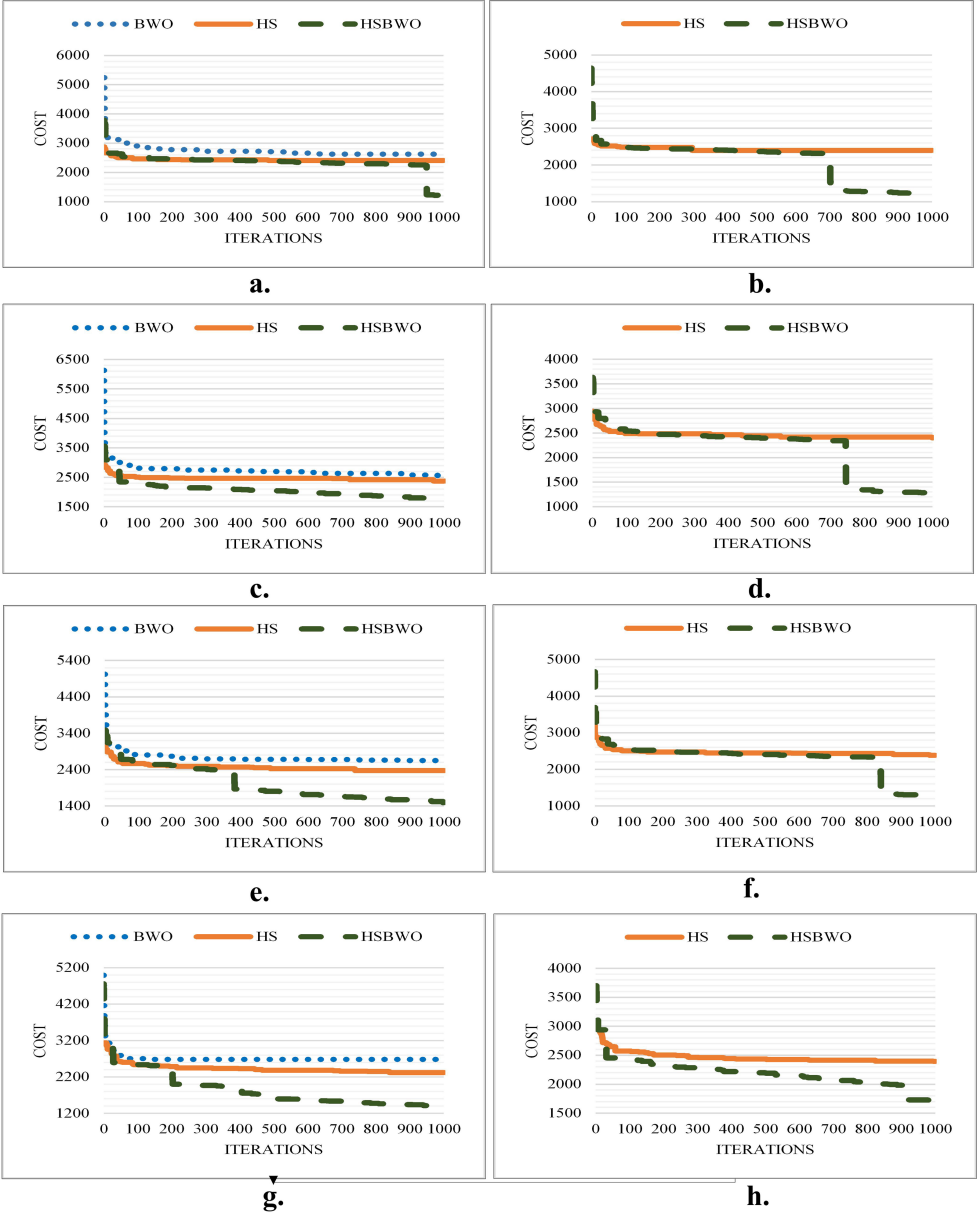
The comparison of convergence rates (population size of 20). (A) Scenario 1. (B) Scenario 2. (C) Scenario 3. (D) Scenario 4. (E) Scenario 5. (F) Scenario 6.

Figure 3 illustrates the best convergence curves for the algorithms under various parameter configurations, showcasing the HSBWO’s capability to navigate the solution space efficiently.

Again, ANOVA statistical analysis was employed to verify the significance of differences between the algorithms:

h0: The average fitness values of the compared algorithms are equal (*i.e., μ*1 = *μ*2 = *μ*3, where *μ* is the mean).

h1: At least one algorithm’s average fitness value is significantly different from the others.

The results, shown in Table 6, confirm significant differences in the mean fitness values, as indicated by *P*-values below 0.05. This reinforces the conclusion that the algorithms differ significantly in performance, leading to the rejection of the null hypothesis (h0) and acceptance of the alternative hypothesis (h1).

#### Simulation results with a population size of 50

As the population size increases to 50, the experimental data in Table 7 continue to validate the dominance of the HSBWO algorithm. It consistently achieves the highest average scores across all tested scenarios (highlighted in bold), demonstrating its scalability and effectiveness. The HS algorithm maintains its second-place performance, further validating the hybrid approach.

**Figure 4:**
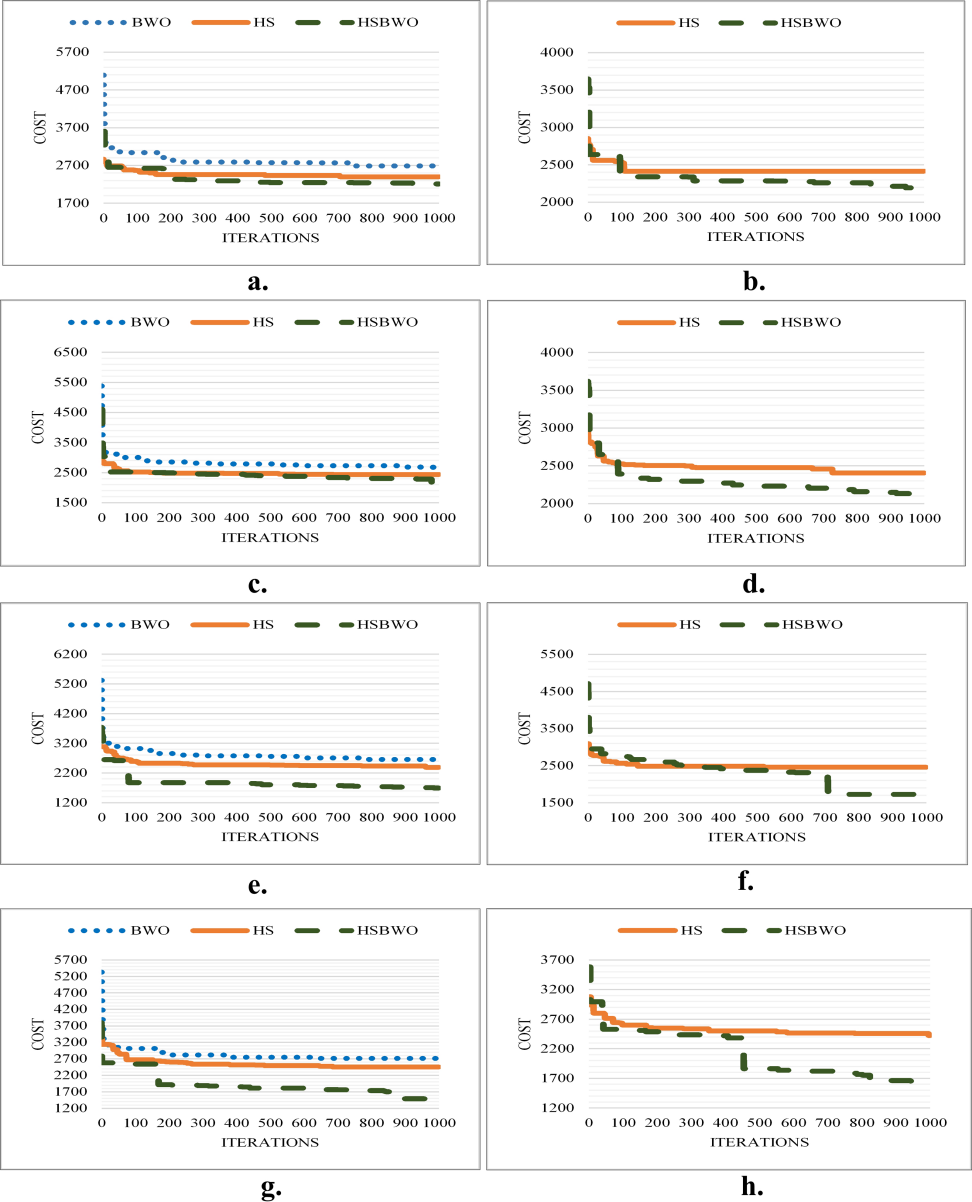
The comparison of convergence rates (population size of 50). (A) Scenario 1. (B) Scenario 2. (C) Scenario 3. (D) Scenario 4. (E) Scenario 5. (F) Scenario 6.

Figure 4 shows the convergence performance for the algorithms across different parameter settings, illustrating the HSBWO’s strong performance in navigating solution space.

To assess the statistical significance of these results, ANOVA analysis was again applied:

h0: The average fitness values of the compared algorithms are equal (*i.e., μ*1 = *μ*2 = *μ*3, where *μ* is the mean).

h1: At least one algorithm’s average fitness value is significantly different from the others.

The ANOVA results in Table 8 indicate significant differences in the mean fitness values, as evidenced by *P*-values below 0.05. This leads to the rejection of the null hypothesis (h0) and supports the alternative hypothesis (h1), confirming that the algorithms produce significantly different fitness results.

**Table 6:** ANOVA test results for the compared algorithms with a 20-population size.

Scenarios	*F*-Value	*P*-Value	*F*-Critical
Sc1	1.04E+02	1.74E−23	3.11E+00
Sc2	4.37E+01	1.51E−08	4.01E+00
Sc3	1.66E+02	2.14E−30	3.10E+00
Sc4	8.78E+01	1.36E−21	3.10E+00
Sc5	1.86E+02	3.47E−32	3.10E+00
Sc6	1.00E+02	2.60E−23	3.10E+00
Sc7	1.35E+02	2.10E−27	3.10E+00
Sc8	9.16E+01	3.91E−22	3.10E+00

**Table 7:** Statistical results of fitness values for 30 experimental tests with a population size of 50.

Scenarios		Sc1	Sc2	Sc3	Sc4	Sc5	Sc6	Sc7	Sc8
HSBWO	***Mean***	**2,277**	**2,258.9**	**2,263.8**	**2,269.8**	**2,192.3**	**2,232.7**	**1,969.8**	**2,198.5**
	***Std***	101.8	87.28	126.3	85.6	226.2	201.8	334.9	245.9
	***Best***	2,066	2,099	1,732	2,131	1,697	1,725	1,490	1,649
	***Worst***	2,432	2,434	2,439	2,434	2,477	2,465	2,500	2,493
HS	***Mean***	2,431.5	2,441.5	2,429.6	2,450	2,417.9	2,446.6	2,417.1	2,449.2
	***Std***	19.58	15.22	19.01	16.25	15.65	21.84	15.72	13.2
	***Best***	2,389	2,398	2,365	2,406	2,390	2,375	2,390	2,422
	***Worst***	2,458	2,470	2,460	2,469	2,454	2,474	2,468	2,471
BWO	***Mean***	2,730.2	–	2,715	–	2,701.4	–	2,700.3	–
	***Std***	17.86	–	18.84	–	24.13	–	21.48	–
	***Best***	2,684	–	2,681	–	2,659	–	2,661	–
	***Worst***	2,761	–	2,762	–	2,762	–	2,749	–

**Notes.**

The best mean results are highlighted in bold font.

#### Simulation results with a population size of 100

Finally, with a population size of 100, the experimental results displayed in Table 9 confirm the superior performance of the HSBWO algorithm. It consistently achieves the highest average fitness scores across all scenarios (highlighted in bold), highlighting its capability to manage larger populations effectively. The HS algorithm also continues to outperform the BWO algorithm, ranking second in all tests.

**Figure 5:**
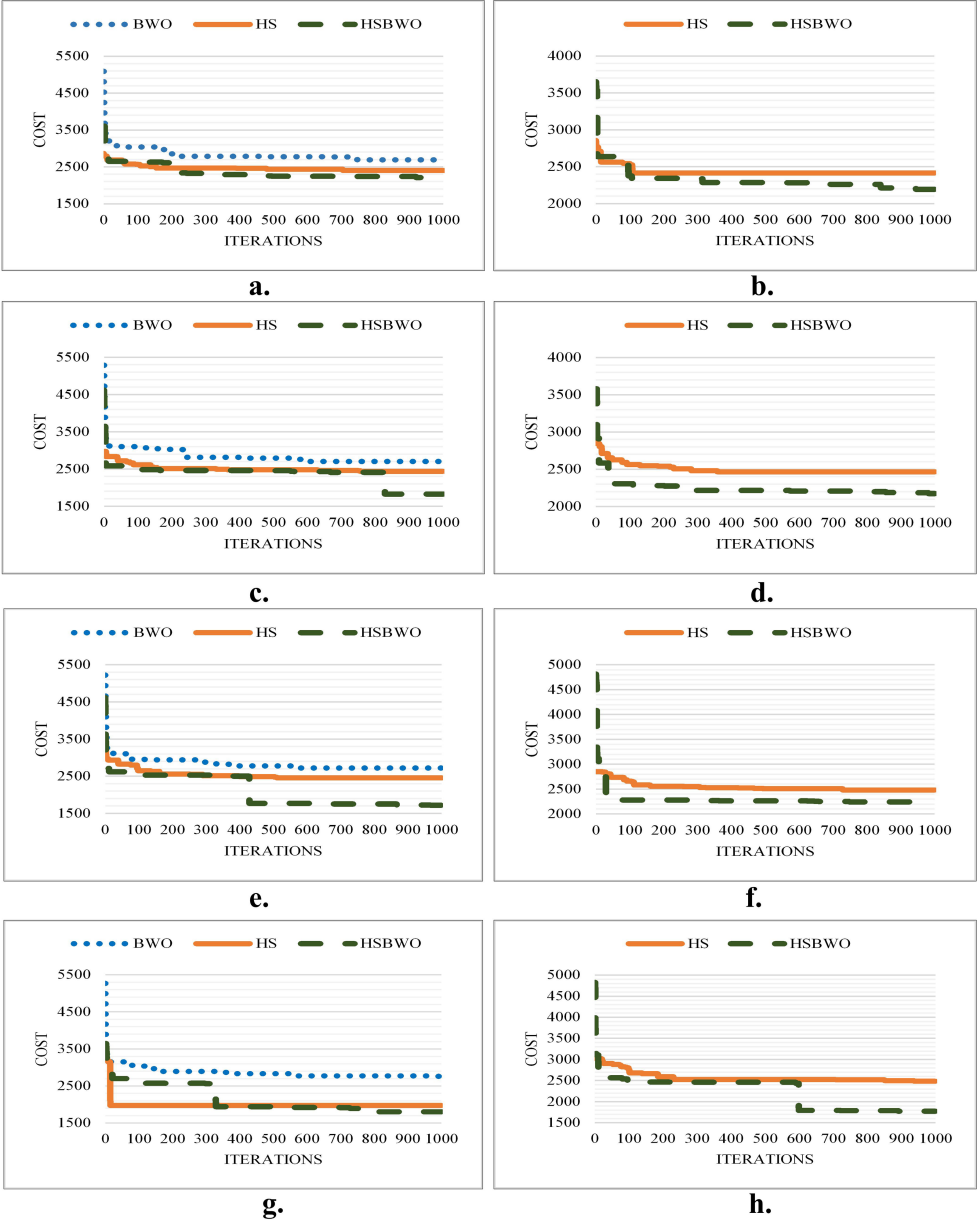
The comparison of convergence rates (population size of 100). (A) Scenario 1. (B) Scenario 2. (C) Scenario 3. (D) Scenario 4. (E) Scenario 5. (F) Scenario 6.

Figure 5 shows the best convergence results of the comparison algorithms across different parameter-setting scenarios.

Furthermore, ANOVA statistical analysis is utilized to check whether the results obtained from the compared algorithms differ significantly, as follows:

h0: The average fitness values of the compared algorithms are equal (*i.e., μ*1 = *μ*2 = *μ*3, where *μ* is the mean).

h1: At least one algorithm’s average fitness value is significantly different from the others.

The ANOVA results in Table 10 demonstrate that the mean fitness values are significantly different from one another since the resulting *P*-values are less than the significance level of 0.05. This indicates that there is a significant difference in the fitness mean results and that not all mean fitness values are equal, leading to the rejection of the null hypothesis (h0) and acceptance of the alternative hypothesis (h1).

**Table 8:** ANOVA test results for the compared algorithms with a 50-population size.

Scenarios	*F*-Value	*P*-Value	*F*-Critical
Sc1	4.57E+02	7.30E−46	3.11E+00
Sc2	1.47E+02	2.71E−17	4.01E+00
Sc3	1.77E+02	1.00E−36	2.71E+00
Sc4	6.61E+02	2.52E−53	3.10E+00
Sc5	4.20E+02	1.99E−45	3.10E+00
Sc6	9.26E+01	2.82E−22	3.10E+00
Sc7	6.69E+02	1.51E−53	3.10E+00
Sc8	9.29E+02	2.00E−59	3.10E+00

**Table 9:** Statistical results of fitness values for 30 experimental tests with a population size of 100.

Scenarios		Sc1	Sc2	Sc3	Sc4	Sc5	Sc6	Sc7	Sc8
HSBWO	***Mean***	**2,363.3**	**2,348.2**	**2,346.1**	**2,352.0**	**2,269.2**	**2,368.1**	**2,032.6**	**2,272.7**
	***Std***	57.913	61.672	116.9	67.7	233.7	64.5	177.6	202.9
	***Best***	2,210	2,193	1,829	2,175	1,722	2,242	1,803	1,772
	***Worst***	2,474	2,455	2,471	2,462	2,517	2,490	2,502	2,510
HS	***Mean***	2,438.1	2,449.8	2,434.1	2,453.7	2,437.5	2,462.4	2,447.1	2,480.5
	***Std***	13.849	16.138	27.56	19.27	21.27	20.385	91.15	24.98
	***Best***	2,402	2,415	2,345	2,375	2,395	2,405	1,978	2,405
	***Worst***	2,461	2,474	2,466	2,482	2,464	2,487	2,499	2,513
BWO	***Mean***	2,767.1	**–**	2,748	–	2,741.3	–	2,728.5	–
	***Std***	30.23	**–**	21.98	–	23.65	–	21.52	–
	***Best***	2,689	–	2,711	–	2,680	–	2,683	–
	***Worst***	2,816	–	2,781	–	2,783	–	2,768	–

**Notes.**

The best mean results are highlighted in bold font.

**Table 10:** ANOVA test results for the compared algorithms with a 100-population size.

Scenarios	*F*-Value	*P*-Value	*F*-Critical
Sc1	8.68E+02	7.80E−57	3.11E+00
Sc2	7.19E+01	1.26E−11	4.01E+00
Sc3	1.66E+02	2.14E−30	3.10E+00
Sc4	8.78E+01	1.36E−21	3.10E+00
Sc5	1.86E+02	3.47E−32	3.10E+00
Sc6	8.03E+01	1.71E−20	3.10E+00
Sc7	1.00E+02	2.60E−23	3.10E+00
Sc8	1.35E+02	2.10E−27	3.10E+00

### Results discussion

The proposed HSBWO algorithm demonstrates superior performance in achieving lower fitness values across various scenarios and population sizes, highlighting its potential for optimizing convergence rates and generating high-quality solutions compared to HS and BWO. The success of the HSBWO algorithm can be attributed to its combination of strong exploration abilities inherited from the HS algorithm and robust exploitation capabilities utilized from the cannibalism process of the BWO algorithm, leading to more accurate and robust scheduling solutions for the transportation problem being optimized. Additionally, the results are further validated by an ANOVA test, which confirmed that the improvements achieved by the HSBWO algorithm are statistically significant.

Notably, in experiments with a population size of 5, the large cost reduction typically happens between 650 to 800 iterations. This is likely because the algorithm may enter a critical phase, where the algorithm transitions from exploration to exploitation, leading to rapid convergence to a feasible area of the solution space.

The effectiveness of the proposed HSBWO algorithm is underscored by the significant percentage improvements it demonstrates. Specifically, for a population size of 5, enhancements over the HS algorithm ranged from approximately 14.2% to 55.2%, while improvements over the BWO algorithm ranged from 37.6% to 56.0%. For population size 20, improvements over HS ranged from 9.7% to 19.4%, and over BWO from 6.4% to 28.1%. For population size 50, improvements over HS ranged from 6.3% to 18.5%, and over BWO from 16.6% to 27.0%. Lastly, for population size 100, improvements over HS ranged from 3.1% to 18.5%, while improvements over BWO ranged from 14.6% to 25.5%. These findings demonstrate that the HSBWO algorithm consistently outperforms both the HS and BWO algorithms across different population sizes and scenarios.

However, it is worth noting that all algorithms exhibit degraded performance in optimizing solutions as population sizes increase. This phenomenon occurs because the search space becomes larger, negatively impacting the convergence speed towards promising areas of the solution space. Furthermore, the results reveal that both HSBWO and HS algorithms perform better under scenario 5 (with settings of 0.9 HMCR, 0.3 PAR, and 0.1 mutation rate) compared to other scenarios, indicating the effectiveness of these parameters in achieving better solution costs. Lastly, despite slight variations observed across different scenarios, all algorithms demonstrate stability in generating their solutions, indicating their reliability and consistency in addressing optimization problems.

## Conclusion

This study addresses the pressing need for enhancing transportation efficiency during mega-events, focusing specifically on the Hajj pilgrimage. Given the diverse transportation and accommodation preferences among pilgrims, finding optimal solutions within a limited timeframe poses a significant challenge. To tackle this challenge, a novel hybrid approach combining the Harmony Search (HS) and Black Widow Optimization (BWO) algorithms, called HSBWO, is proposed. The HS algorithm has strong exploration ability but has difficulties in exploiting the search space; on the other hand, the BWO algorithm has strong exploitation ability but may encounter early convergence. To efficiently utilize the strengths of both algorithms, the proposed approach incorporates the cannibalism mechanism of the BWO algorithm into the improvisation process of the HS algorithm. The proposed HSBWO algorithm demonstrates superior performance in achieving lower fitness values across various scenarios and population sizes, highlighting its potential for optimizing convergence rates and generating high-quality solutions compared to the HS and BWO algorithms. The HSBWO algorithm demonstrated superior performance, with improvements in average fitness values ranging from 3.62% to 22.7% over HS and from 14.6% to 37.1% over BWO, depending on the specific scenarios and population sizes used.

In addition, the powerful exploration and exploitation capabilities of the HSBWO algorithm can be extended beyond transportation scheduling optimization to various potential applications in many optimization domains, including:

• Logistics and supply chain optimization: This covers inventory control, route planning, and warehouse operations, where effective resource allocation and scheduling are crucial.
• Energy management systems: Optimizing load demand balancing, scheduling of energy resources, and optimizing energy distribution to improve transmission efficiency and minimize losses.
• Healthcare resource scheduling: Including scheduling medical staff, operating rooms, and patient appointments, minimizing waiting times for patients and making efficient use of available resources.
• Manufacturing and production planning: This includes solving job shop scheduling and optimizing production lines to minimize cost while meeting deadlines and quality requirements.

Although the proposed HSBWO algorithm offers significant advantages in terms of enhanced local search capability and practical application to complex optimization problems, it also has several drawbacks, such as increased computational complexity and sensitivity to parameter tuning, which require further research in future work.

##  Supplemental Information

10.7717/peerj-cs.2526/supp-1 Supplemental Information 1 Simulation Results

10.7717/peerj-cs.2526/supp-2 Supplemental Information 2 Optimization algorithm Matlab Code

## Additional Information and Declarations

### Data Availability

The following information was supplied regarding data availability: The statistical analysis of the experiments and code are available in the Supplemental Files.

## References

[ref-1] Abu-Hashem MA, Shambour M (2024). An improved black widow optimization (IBWO) algorithm for solving global optimization problems. International Journal of Industrial Engineering Computations.

[ref-2] Al-Sabban SA, Ramadan HM (2005). A simulation study of the shuttle-bus pilgrim transportation system between the holy sites for the 1422H Hajj season. King AbdulAziz University, Engineering Sciences.

[ref-3] Alia OMD, Mandava R (2011). The variants of the harmony search algorithm: an overview. Artificial Intelligence Review.

[ref-4] Elkhouly R, Tamaki E, Iwasaki K (2023). Mitigating crowded transportation terminals nearby mega-sports events. Behaviour & Information Technology.

[ref-5] Felemban E, Fatani A, Rehman FU (2019). An optimized scheduling process for a large crowd to perform spatio-temporal movements safely during pilgrimage.

[ref-6] Felemban E, Qahtani K, Hawsawi A, Shahri A (2017). Crowd simulation model for crowd movement in the Holy Mosque.

[ref-7] Geem ZW, Kim JH, Loganathan GV (2001). A new heuristic optimization algorithm: harmony search. Simulation.

[ref-8] Haase K, Kasper M, Koch M, Müller S (2019). A pilgrim scheduling approach to increase safety during the Hajj. Operations Research.

[ref-9] Hayyolalam V, Pourhaji Kazem AA (2020). Black widow optimization algorithm: a novel meta-heuristic approach for solving engineering optimization problems. Engineering Applications of Artificial Intelligence.

[ref-10] Huang ZM, Chen WN, Li Q, Luo XN, Yuan HQ, Zhang J (2020). Ant colony evacuation planner: an ant colony system with incremental flow assignment for multipath crowd evacuation. IEEE Transactions on Cybernetics.

[ref-11] Hussain O, Felemban E, Rehman FU (2021). Optimization of the Mashaer shuttle-bus service in Hajj: Arafat-Muzdalifah case study. Information 2021.

[ref-12] Khan EA, Shambour MK (2023). An optimized solution for the transportation scheduling of pilgrims in Hajj using harmony search algorithm. Journal of Engineering Research.

[ref-13] Liao XC, Chen WN, Guo XQ, Zhong J, Hu XM (2023). Crowd management through optimal layout of fences: an ant colony approach based on crowd simulation. IEEE Transactions on Intelligent Transportation Systems.

[ref-14] Lu W, Zhang Y, Li P, Wang T (2023). Estimating urban rail transit passenger inflow caused by special events occurrences fusing multi-source data. Neural Computing and Applications.

[ref-15] Madhiarasan M, Cotfas DT, Cotfas PA (2023). Black widow optimization algorithm used to extract the parameters of photovoltaic cells and panels. Mathematics.

[ref-16] Morgan AA, Khayyat KMJ (2022). Improving emergency services efficiency during Islamic pilgrimage through optimal allocation of facilities. International Transactions in Operational Research.

[ref-17] Qin F, Zain AM, Zhou KQ (2022). Harmony search algorithm and related variants: a systematic review. Swarm and Evolutionary Computation.

[ref-18] Rehman F, Felemban E (2019). A preference-based interactive tool for safe rescheduling of groups for hajj.

[ref-19] Shambour MKY (2018). Vibrant search mechanism for numerical optimization functions. Journal of Information and Communication Technology.

[ref-20] Shambour MK, Khader AT, Abusnaina AA, Shambour Q (2014). Modified tournament harmony search for unconstrained optimisation problems. Advances in Intelligent Systems and Computing.

[ref-21] Shambour MK, Khan E, Salibi A (2017). Distribute Mina camps automatically to increase the capacity and efficiency.

[ref-22] Shambour MK, Khan E (2019). A heuristic approach for distributing pilgrims over Mina Tents. Journal of King Abdulaziz University.

[ref-23] Yasein MS, Khan EA (2023). Optimizing Shuttle-Bus Systems in Mega-Events using Computer Modeling: A Case Study of Pilgrims’ Transportation System. International Journal of Advanced Computer Science and Applications (IJACSA).

